# Congenital hemidysplasia with ichthyosiform naevus and limb defects (CHILD) syndrome without hemidysplasia

**DOI:** 10.1111/bjd.13636

**Published:** 2015-05-28

**Authors:** A. Estapé, D. Josifova, D. Rampling, M. Glover, V.A. Kinsler

**Affiliations:** ^1^ Department of Paediatric DermatologyGreat Ormond Street Hospital for ChildrenLondonWC1N 3JHU.K; ^2^ Department of GeneticsGuy's HospitalLondonU.K; ^3^ Department of Paediatric HistopathologyGreat Ormond Street Hospital for ChildrenLondonWC1N 3JHU.K; ^4^ Genetics and Genomic MedicineUCL Institute of Child HealthLondonU.K

dear editor, Congenital hemidysplasia with ichthyosiform naevus and limb defects (CHILD) syndrome is a rare X‐linked dominant disorder caused by mutations in *NSDHL*.^1^, ^2^ This gene encodes the enzyme 3β‐hydroxylsterol dehydrogenase, which catalyses a step in the cholesterol biosynthetic pathway.^1^ Characteristic signs present at birth or in the first weeks of life, namely strikingly unilateral ichthyosiform skin lesions with a sharp midline demarcation, and ipsilateral limb defects (ranging from hypoplasia of the phalanges to absence of the entire extremity).^1^, ^3^ The face is usually spared. The central nervous system, lungs, heart and kidneys can also be involved.^2^ We report a case of CHILD syndrome without the characteristic hemidysplasia.

**Figure 1:**
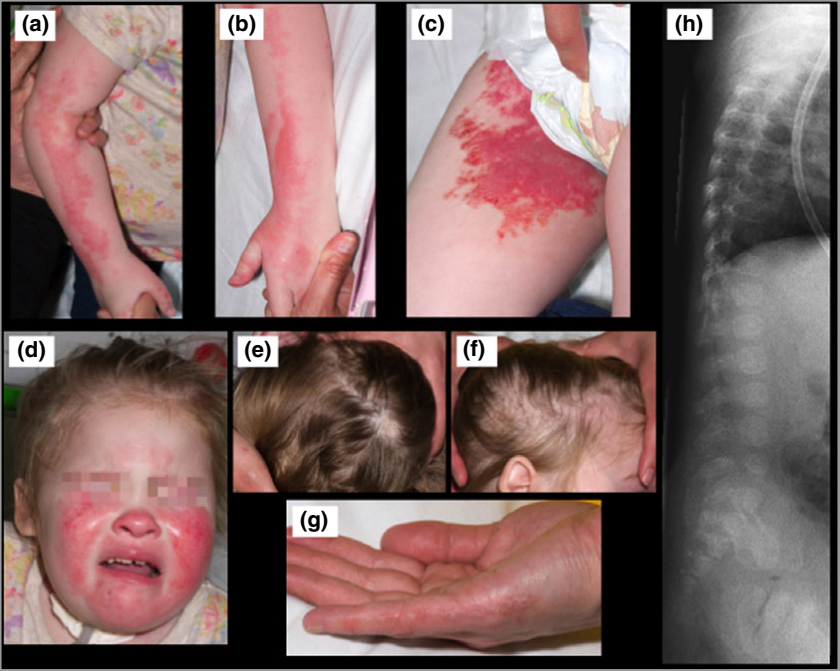
(a–d) Clinical and radiological images. Large erythematous xerotic plaques in a linear distribution on both upper limbs, affecting the right groin and thigh, and diffusely bilaterally on the cheeks. (e, f) Small areas of nonscarring alopecia bilaterally on the scalp. (g) Linear area of erythema and hyperkeratosis on the mother's right hand. (h) A lateral view of the spine showing punctate calcifications in the vertebrae, typical of chondrodysplasia punctata.

A 7‐year‐old girl was referred to our department for evaluation of areas of persistently inflamed and hyperkeratotic skin. She was born at 35 weeks of gestation by caesarean section for breech presentation, without gross limb defects. Her medical history revealed a radiological diagnosis of spinal chondrodysplasia punctata with atlantoaxial subluxation and intermittent spinal compression, bilateral hip subluxation, severe neurodevelopmental delay, chronic lung disease requiring tracheostomy and night‐time ventilation, gastro‐oesophageal reflux and gastrostomy feeding. Brain magnetic resonance imaging had revealed general loss of white matter bulk but with no asymmetry. The skin lesions had been present from birth, and persistent symptoms were recurrent painful fissuring and pruritus. Cutaneous examination revealed inflammatory ichthyotic lesions along the lines of Blaschko on both upper limbs (right more than left), in the right groin, and diffusely on both cheeks. In addition, there were areas of persistent nonscarring alopecia bilaterally on the scalp, one circular and well circumscribed, and the other more diffuse, with no evidence of trichotillomania. Dysmorphic facial features included deep‐set eyes and epicanthic folds. Importantly, and compatible with previous reports,^4^ the patient's mother had similar but much milder linear hyperkeratotic skin lesions affecting the right arm and hand, but was otherwise unaffected (Fig. 1).

**Figure 2:**
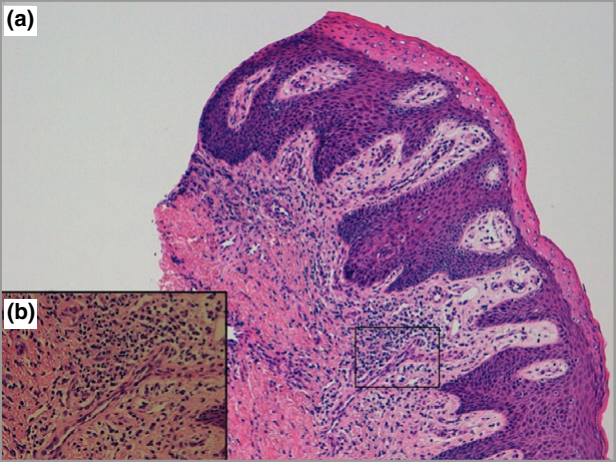
(a, b) Skin biopsy showing features compatible with ichthyosiform dermatosis: acanthosis and extension of rete pegs of the epidermis, marked parakeratotic scaling with loss of the granular layer, occasional clusters of neutrophils, and perivascular and dermal lymphohistiocytic infiltrates. No verrucous xanthoma was seen. (a) Magnification 10×; (b) magnification 40×.

Skin biopsy of affected skin from the patient showed features compatible with ichthyosiform dermatosis: acanthosis and extension of rete pegs of the epidermis, marked parakeratotic scaling with loss of the granular layer, occasional clusters of neutrophils, and perivascular and dermal lymphohistiocytic infiltrates (Fig. 2), but without the characteristic verruciform xanthoma of CHILD syndrome. Array comparative genomic hybridization analysis of peripheral blood leucocyte DNA was normal, excluding large copy number changes (resolution 150 kb). Sanger sequencing revealed a novel germline heterozygous microdeletion inducing a frameshift and premature stop codon at position 119 of a total translated length of 373 amino acids in *NSDHL* (c.357_358del, p.Arg119Serfs*1d), predicted damaging *in silico*. This mutation is compatible with the previously reported disease‐causing mutations in classical CHILD syndrome,^1^ and on the basis of current knowledge confirmed a genetic diagnosis of CHILD syndrome in both the patient and her mother. No other changes in the gene were identified. Previously reported mutations have principally been heterozygous nonsense or missense point mutations, and large heterozygous deletions have been described.^3^, ^5^ This novel microdeletion would be predicted to cause loss of function by truncating the protein before the catalytic site of the gene product.

Traditionally, management of skin lesions in CHILD syndrome has been difficult; however, Paller *et al*. recently reported an innovative and highly successful topical therapy in two patients – co‐application of cholesterol 2% and lovastatin 2% – based on the role of NSDHL in cholesterol metabolism.^6^ These results have been replicated in three further reports of patients using co‐application of cholesterol 2% and simvastatin 2%,^7^ and has been successfully trialled in our patient, with complete resolution of erythema and hyperkeratosis in the treated groin area.

Two other conditions are interesting to consider in this atypical case (Table 1). Firstly, the recently described allelic disorder CK syndrome (MIM #300831), caused by milder mutations in *NSDHL*, is characterized by cortical malformations, typical facial features, asthenic body habitus and no described cutaneous phenotype. Our patient does not exhibit typical features of this syndrome. Secondly, Conradi–Hunermann–Happle (CHH) syndrome is an X‐linked dominant disorder caused by mutations in the emopamil‐binding protein gene (*EBP*), which governs the next step in the cholesterol biosynthetic pathway after *NSDHL*. The presence of chondroplasia punctata, intellectual disability and alopecia have all been described in CHH syndrome; however, disproportionate skeletal growth, growth deficiency, characteristic linear/whorled pigmentary lesions and cataracts were lacking in our patient. Furthermore, the histological features in this case support a diagnosis of CHILD syndrome and exclude a diagnosis of CHH.

**Table 1:** Comparison of features of congenital hemidysplasia with ichthyosiform naevus and limb defects (CHILD) syndrome, Conradi–Hunermann–Happle (CHH) syndrome, CK syndrome and our patient

Syndrome	CHILD	CHH	CK	Our patient
Genetics	X‐linked dominant disorder caused by heterozygous loss of function mutations in *NSDHL*	X‐linked dominant disorder caused by mutations in *EBP*	X‐linked recessive disorder caused by milder mutations in *NSDHL*	Heterozygous microdeletion inducing a frameshift and premature stop codon in *NSDHL*
Cutaneous phenotype	Unilateral ichthyosiform skin lesions with sharp midline demarcation	Ichthyosiform erythroderma	None described	Inflammatory linearichthyotic lesions on both upper limbs, right groin and diffusely bilaterally on cheeks
	Psychotropic skin lesions	Linear or whorled pigmentary lesions		Areas of nonscarring alopecia
		Striated ichthyosiform hyperkeratosis		
		Patchy cicatricial alopecia		
Histopathological features	Verruciform xanthoma	Hyperkeratosis and acanthosis	–	Acanthosis and extension of rete pegs
	Hyperkeratosis, parakeratosis and acanthosis	Calcium deposits within the stratum corneum		Marked parakeratotic scaling with loss of the granular layer
	Inflammatory and lipid‐laden infiltrated within the dermal papillae	Focal pigmentation of basal layer		Occasional clusters of neutrophils
				Perivascular and dermal lymphohistiocytic infiltrates
Associated defects	Congenital hemidysplasia	Chondrodysplasia punctata	Cortical malformations	Spinal chondrodysplasia punctata
	Ipsilateral limb defects	Intellectual disability	Characteristic craniofacial features	Bilateral hip subluxation
	Visceral malformations	Short stature	Asthenic body habitus	Severe neurodevelopmental delay
	CNS anomalies	Cataracts	Behaviour problems	Chronic lung disease
				Gastro‐oesophageal reflux

CNS, central nervous system.

A clear bilateral presentation in CHILD syndrome has been reported rarely before, once with characteristic skin lesions affecting the body folds in a near‐symmetrical distribution, associated with a novel missense mutation in *NSDHL*,^8^, ^9^ twice with contralateral linear skin lesions,^1^ once with bilateral, almost symmetrical, linear lesions on the extremities.^10^ Our case confirms this bilateral cutaneous presentation, emphasizes the significant inter‐ and intrafamilial variation, and extends the noncutaneous phenotype of CHILD syndrome.
